# How Social Norms Influence Purchasing Intention of Domestic Products: The Mediating Effects of Consumer Ethnocentrism and Domestic Product Judgments

**DOI:** 10.3390/bs13060453

**Published:** 2023-05-31

**Authors:** Qifan Jia, Sizhe Zhou, Run Liu, Yihan Zuo, Cuiyu Pan, Yu Chen, Yiyue Gong, Rui Chen

**Affiliations:** 1 School of Journalism, Communication University of China, Beijing 100024, China; lr0613@cuc.edu.cn (R.L.); zzuoyihan@cuc.edu.cn (Y.Z.); pcy@cuc.edu.cn (C.P.); chenyu0409@cuc.edu.cn (Y.C.); gongyy@cuc.edu.cn (Y.G.); 2 Department of Psychological and Behavioural Science, London School of Economics and Political Science, London WC2A 2AE, UK; s.zhou28@lse.ac.uk

**Keywords:** social norms, consumer ethnocentrism, domestic product judgments, domestic purchasing intention, China

## Abstract

Buying domestic products has become increasingly important in many countries. As a form of social influence, social norms affect people’s domestic purchasing intentions and behavior. The current study aims to examine the mechanisms by which social norms influence domestic purchasing intentions through the lens of consumer ethnocentrism and domestic product judgments. The data were collected through an online survey in China, and a total of 346 valid responses were obtained. The results indicate that social norms influence domestic purchasing intention through four paths, namely, direct path, motivational path, cognitive path, and motivational–cognitive path. Consumer ethnocentrism and domestic product judgments, serving as the motivational and cognitive factors, respectively, play mediating and serial mediating roles in the relationship between social norms and domestic purchasing intention. In addition, consumer ethnocentrism has two dimensions, namely, pro-domestic and anti-foreign consumer ethnocentrism, and only the former plays a significant role in the model. The current study has theoretical contributions to research on domestic purchasing intention and practical implications for interventions in domestic purchasing behavior. Future studies are encouraged to conduct experiments, distinguish between different types of social norms, measure purchasing behavior, and verify the relationships in other countries.

## 1. Introduction

With the rise of anti-globalization, regional protectionism, and nationalist discourse worldwide, there has been a growing trend of defending domestic products and companies. For instance, in 2017, former President Donald Trump signed an executive order called “Buy American, Hire American”, aimed at supporting domestic products and companies [1]. Similarly, in 2021, the incumbent president Biden signed an executive order titled “Ensuring the Future is Made in All of America by All of America’s Workers” [2]. Many other countries, including Australia, Indonesia, Vietnam, South Africa, and China, have also launched similar campaigns to promote domestic products and companies [3].

Given the great importance of promoting domestic products, many studies have investigated the influencing factors of domestic purchasing intention and behavior, including product characteristics [4], consumer demographics [5], consumer characteristics [6], and cultural factors [7]. However, there is still a research gap in understanding the role that social influence plays in this.

Social norms, which refer to the rules and standards that are shared by members of a group, are the most typical social influence [8]. In this research, social norms refer to the perception, attitudes, and behaviors about domestic purchasing that are approved of and expected by the majority of people in society. Previous studies have confirmed that social norms have an impact on domestic purchasing intention and behavior [9,10,11]. However, no study has investigated the mechanism of the effect. The current study aims to examine the mechanisms of social norms’ effect on domestic purchasing intention. Based on previous research showing that social norms affect people’s behavior in four ways, we come up with the four paths through which social norms influence domestic purchasing intention, i.e., the direct path, the motivational path, the cognitive path, and the motivational–cognitive path. Consumer ethnocentrism and domestic product judgments represent the motivational factor and cognitive factor, respectively.

This research employed a questionnaire survey to measure variables and path analysis and a mediation test to examine the data. The advantage of this method is that it not only provides information about the direct relationships between variables but also enables the examination of indirect effects. Additionally, simultaneously including different paths in the model allows for mutual control of the variables. In the current study, the direct effect of social norms on domestic purchasing intention and the mediating effects and serial mediating effect of consumer ethnocentrism and domestic product judgments on the relationship between social norms and domestic purchasing intention are examined.

The current study is the first empirical investigations to explore the mechanisms of social norms’ effect on domestic purchasing intention. Moreover, coming up with the four paths through which social norms influence domestic purchasing intention and simultaneously examining them in the same model is a novelty. This research can fill the current gap in knowledge on how domestic consumption is affected by social influence and help policy makers and marketers to develop targeted strategies and interventions to promote domestic consumption.

This research takes China as the research object, because China has a large consumer market which plays an important role in the world economy; people in China accept the idea that domestic purchasing is the socially desirable way of consumption [12]; and social norms about purchasing domestic products have an impact on people’s consumption behavior [13]. It is worth mentioning that although the specific objectives of the campaigns promoting domestic products may vary across countries, encompassing economic, social, and environmental dimensions, they can all play a role in domestic purchasing intention and behavior through social norms.

## 2. Literature Review

### 2.1. The Four Paths through Which Social Norms Affect Domestic Purchasing Intention

Social norms are important to the normal operation of society and the effective conduct of social cooperation. In the evolution and development of human society, social norms as an informal system without the force of laws constrain a wide variety of anti-social behavior, such as discrimination [14] and corruption [15,16], and promote various pro-social behavior, such as fair behavior [17] and altruistic behavior [18].

Based on previous studies, social norms affect people’s behavior in four ways. First, by direct influence: people unconsciously conform to the majority under social influence [19]. Second, by changing people’s motivation: Social norms convey the value orientation of most people in society. Individuals tend to internalize external social norms through social learning to guide their behavior [20]. Third, by changing people’s cognition: To pursue the correct decision making, individuals consciously or unconsciously take other people’s behaviors or opinions as reference [21]. If most people in society behave in a certain way, people will think that the behavior is more reasonable, and then produce the corresponding behavior. Fourth, by changing people’s motivations and cognition successively: If urges, drives, and wants are activated, the cognitive process can be biased to meet the need. The internalized social norms will influence people’s cognitions, which in turn influence behavior [22]. Domestic purchasing intention can be affected by social norms through the four paths.

### 2.2. The Direct Path: The Direct Effect of Social Norms on Domestic Purchasing Intention

According to the theory of planned behavior, behavioral intention is the most proximal determinant of people’s behavior, and it is shaped by attitude, social norms (termed subjective norms in the theory), and perceived behavioral control [19]. Among the three constructs, social norms explain the social influence on human behavior.

Previous studies have confirmed the effect of social norms on the purchasing intention of a variety of products, including utilitarian products [23], luxury products [24], fair trade products [25], organic products [26], and so on. Specific to domestic products, Granzin and Painter find that social norms significantly predict consumers’ domestic purchasing behavior in Portugal and the United States (the US) [9]. Maduku and Phadziri find that social norms are significantly correlated with consumers’ domestic purchasing bias in South Africa [11]. Jia et al. find that social norms are positively related to consumers’ willingness to buy domestic products in China [9].

### 2.3. The Motivational Path: The Mediating Effect of Consumer Ethnocentrism on the Relationship between Social Norms and Domestic Purchasing Intention

Consumer ethnocentrism is an important concept to explain cross-national product choice. It refers to the belief held by consumers about the appropriateness and morality of buying foreign products [27]. People with high consumer ethnocentrism believe that buying foreign products damages the domestic economy, and even leads to the unemployment of compatriots, which is inappropriate and immoral [3]. Social norms and consumer ethnocentrism are both normative beliefs about the appropriateness and morality of buying foreign or domestic products. However, consumer ethnocentrism refers to personal norms [27], which are different from social norms. Social norms reflect external rules, while personal norms reflect internal standards [28].

It has been argued that personal norms are internalized social norms [29]. According to social cognition theory, people acquire values of what is right and what is wrong through social learning [30]. Specifically, social norms convey a signal that purchasing a certain type of product is desirable, which will form people’s personal norms about the correctness of purchasing this type of product. Abundant studies have shown that social norms about purchasing a certain type of product shape personal norms [25,26,31,32]. Research on domestic purchasing also shows that there is a significant positive relationship between social norms and personal norms (consumer ethnocentrism; [9,11]).

According to norm activation theory, personal norms are the driving force of altruistic behavior [33]. Domestic purchasing can be seen as altruistic, especially in less-developed countries, where foreign products are often of higher quality and people need to sacrifice their interests to favor domestic ones [34]. Meta-analysis research shows that consumer ethnocentrism is positively correlated with domestic purchasing intention [35]. This relationship exists in many counties, such as the US [36], Spain [37], India [6], and China [38,39,40].

### 2.4. The Cognitive Path: The Mediating Effect of Domestic Product Judgments on the Relationship between Social Norms and Domestic Purchasing Intention

Social norms can alter people’s cognition. Domestic product judgments, which are also termed quality perception, quality judgment, and general beliefs toward domestic products, refer to evaluations of domestic products’ quality, price, reliability, value for the money, etc. [41].

Attribution theory suggests that people make causal explanations for what happens to predict and control the environment [42]. Social norms send a message about the choices most people currently make, i.e., most people in society prefer domestic products. Previous research shows that social norms are negatively associated with the judgment of products from foreign countries [43]. However, to our knowledge, no research has examined the effect of social norms on domestic product judgments. Given the literature above, we assume that higher social norms may lead to more positive evaluations of domestic products because people might attribute the popularity of domestic products to their higher quality.

According to the hypothesis of the economic man, people are rational and self-interested, and they act in a way that maximizes utility in economic activities [44]. Product judgments play important roles in consumer choice, i.e., if people give better evaluations on the products, they are more inclined to buy them [45]. Zebal and Jackson find that one of the incentives for Bangladeshi consumers to buy local clothing brands is positive product judgment [4]. Rahnama finds that Iranian consumers are willing to buy domestic rice because of its good quality and price [46]. More direct evidence shows that domestic product judgments are positively correlated with willingness to buy domestic products [41,47].

### 2.5. The Motivational–Cognitive Path: The Serial Mediating Effect of Consumer Ethnocentrism and Domestic Product Judgments on the Relationship between Social Norms and Domestic Purchasing Intention

Social identity theory claims that people are motivated to identify themselves as a member of groups and develop an in-group preference to maintain a positive self-identity [48]. When people view a group as their in-group, they will not only be favorably biased toward the in-group members, but also the products of the in-group [22]. Shimp and Sharma find that people with high consumer ethnocentrism have a halo effect on domestic products; that is, compared with people with low consumer ethnocentrism, they have more positive evaluations of domestic products [27].

Many studies confirm Shimp and Sharma’s conclusion [27]. For example, Brodowsky finds that consumers with higher consumer ethnocentrism in the US have more positive evaluations of cars designed or manufactured and assembled in the US [49]. Orth and Firbasova find that Czech consumers with higher consumer ethnocentrism rate domestic yogurt higher [50]. The positive relationship between consumer ethnocentrism and domestic product judgments is also found in Poland [51], China [52], Austria and Slovenia [47], the United Kingdom [53], and Slovakia [54].

### 2.6. The Two Dimensions of Consumer Ethnocentrism

Since Shimp and Sharma proposed the concept of consumer ethnocentrism and its measurement (the CETSCALE) [27], it has been examined in different countries. Although most of them agreed that consumer ethnocentrism is a single-dimensional construct, some studies confirmed a two-dimensional construct. For example, Akbarov uses an Azerbaijani sample and finds that consumer ethnocentrism has two dimensions, i.e., “hard consumer ethnocentrism”, which contains a strong hostile attitude toward foreign products; and “soft consumer ethnocentrism”, which does not prompt exclusions of foreign products but simply emphasizes the preference for domestic products [5]. This is consistent with studies conducted in Greece [55] and Malaysia [56]. In addition, studies on Chinese consumers obtain similar results. For instance, Wei et al. defines the two dimensions as “pro-China ethnocentrism” and “pro-foreign ethnocentrism” [57], Hsu and Nien define the two dimensions as “conservative patriotism” and “defensive patriotism” [58], and Bi et al. just define the two dimensions as “CE1” and “CE2” [59]. According to the connotation of these dimensions, we name the two dimensions “pro-domestic consumer ethnocentrism” and “anti-foreign consumer ethnocentrism”.

Previous studies show that the two dimensions have distinct effects on purchasing intention and product judgments. For example, Hsu and Nien demonstrate that conservative patriotism (pro-domestic consumer ethnocentrism) has a great impact on domestic purchasing intention, while defensive patriotism (anti-foreign consumer ethnocentrism) does not among Chinese consumers [58]. Teo et al. find that the path from consumer ethnocentrism to perception towards domestic brands in Malaysia is significant for soft (pro-domestic) consumer ethnocentrism but not significant for hard (anti-foreign) consumer ethnocentrism [56].

### 2.7. The Hypotheses of the Research

The current study aims to examine the mechanisms of social norms’ effect on domestic purchasing intention through the four paths. Given the literature above, we propose that

**H1:**  *Social norms influence domestic purchasing intention through the direct path. Specifically, social norms have a direct effect on domestic purchasing intention*.

**H2:**  *Social norms influence domestic purchasing intention through the motivational path. Specifically, pro-domestic consumer ethnocentrism mediates the relationship between social norms and domestic purchasing intention, while anti-foreign consumer ethnocentrism does not*.

**H3:**  *Social norms influence domestic purchasing intention through the cognitive path. Specifically, domestic product judgments mediate the relationship between social norms and domestic purchasing intention*.

**H4:**  *Social norms influence domestic purchasing intention through the motivational–cognitive path. Specifically, pro-domestic consumer ethnocentrism and domestic product judgments mediate the relationship between social norms and domestic purchasing intention sequentially, while anti-foreign consumer ethnocentrism and domestic product judgments do not.*

**Figure 1:**
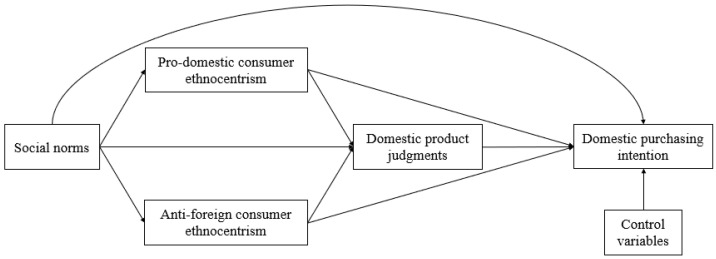
The research hypotheses.

See Figure 1 for the research hypotheses.

## 3. Materials and Methods

### 3.1. Sample and Data Collection

The data were from the same sample as Jia et al.’s study 2 [9], which was collected in 2022 through an online survey by sending questionnaire links on WeChat. A total of 512 Chinese consumers over 18 years old finished the questionnaire, and 346 valid responses were obtained. Among them, 54% were females. Respondents ranged in age from 18 to 79, with an average of 35 years old. See Table 1 for demographic information on the respondents.

In the process of data collection, all the participants were informed about the purpose of the study, and they were informed that the data would be used only for scientific research, their participation in the survey was completely voluntary, and they could choose to withdraw at any time.

### 3.2. Instruments

The questionnaire includes three parts. First is the single-item domestic purchasing intention measurement, which was adapted from Tong and Li’s research [52]. Second is the influencing factors measurements, including 3-item social norms scale, which was the same as Jia et al.’s research [9]; 17-item consumer ethnocentrism scale, which was from Shimp and Sharma’s CETSCALE [27]; and 3-item domestic product judgements scale, which was adapted from Kervyn et al.’s research [60]. Third is the demographic information measurements, including gender, age, education level, and average monthly income. All the items except for demographics were measured on a seven-point Likert scale (1 = totally disagree; 7 = totally agree). See Table 2 for the items of the constructs.

## 4. Results

### 4.1. Common Method Bias

We adopted confirmatory factor analysis (CFA) to test for common method bias [61]. We found that the fit of the three-factor model was significantly better than that of the single-factor model (Δ*χ*^2^ = 718.23, Δ*df* = 3, *p* < 0.01). Additionally, the fit indices of the three-factor model were not different from those of the measurement model with an unmeasured latent variable (ΔCFI = 0.07 < 0.10, ΔTLI = 0.07 < 0.10, ΔRMSEA = 0.02 < 0.05). Therefore, common method bias was not a problem in the current research.

### 4.2. Descriptives and Correlations

Table 3 shows the descriptive statistics and correlations of the variables. We found that social norms (*M* = 5.70, *SD* = 0.95), pro-domestic consumer ethnocentrism (*M* = 4.78, *SD* = 1.36), domestic product judgments (*M* = 5.20, *SD* = 1.24), and domestic purchasing intention (*M* = 5.87, *SD* = 1.13) were higher than the midpoint 4, while anti-foreign consumer ethnocentrism was lower than 4 (*M* = 3.55, *SD* = 1.53). All of them were significantly positively correlated with each other (*r*s = 0.38–0.80, *p*s < 0.01).

### 4.3. Measurement Model

To test the dimensionality of the CETSCALE, we adopted an exploratory factor analysis (EFA) with SPSS 27.0. Kaiser–Meyer–Olkin (KMO) and Bartlett tests were conducted to examine the suitability of EFA for the data. The results showed that KMO = 0.96 > 0.50, and Bartlett’s test of sphericity was significant (*p* < 0.01), which indicated that EFA was suitable for the data. Then, a principal components analysis (with varimax rotation) was conducted to extract the factors. The results suggested a two-factor solution, which explained 67.68% of the variance. Item 10 was deleted because its loadings on both factors were greater than 0.50. The first dimension included eight items (pro-domestic consumer ethnocentrism: CE1, CE2, CE3, CE4, CE7, CE8, CE9, and CE13), and the second dimension included eight items (anti-foreign consumer ethnocentrism: CE5, CE6, CE11, CE12, CE14, CE15, CE16, and CE17).

To test the reliability and validity of these constructs, we conducted a confirmatory factor analysis with Mplus 7.4. The results showed that the model had a good fit, *χ*^2^ = 638.01, *df* = 203, *χ*^2^/*df* = 3.14. CFI = 0.92, TLI = 0.91, RMSEA = 0.08, SRMR = 0.05. The factor loadings of the items were higher than the recommended threshold of 0.60 [62], except for CE3, which had a factor loading of 0.49 and was deleted in the model. See Table 2 for details. The values of Cronbach’s α and composite reliability were above 0.70, which indicated good reliability of the scales [63]. The average variance extracted (AVE) values were above 0.50 [64], which indicated adequate convergent validity. The square root of the AVE score of each construct was greater than its correlation coefficients with other constructs (except that the square root of the AVE score of pro-domestic consumer ethnocentrism was slightly lower than the correlation between pro-domestic consumer ethnocentrism and anti-foreign consumer ethnocentrism), and the heterotrait–monotrait (HTMT) ratios were below the threshold value of 0.90, which indicated acceptable discriminant validity [64,65]. See Table 3 and Table 4 for details. Above all, the reliability and validity of the constructs were satisfied and suitable for examining the structural model.

### 4.4. Structural Model

To test the structural model, we conducted a path analysis with Mplus 7.4, and the results are presented in Table 5. The results showed that the model had a good fit, *χ*^2^ = 51.09, *df* = 12, *χ*^2^/*df* = 4.26. CFI = 0.96, TLI = 0.91, RMSEA = 0.10, SRMR = 0.06. We found that social norms were positively related to domestic purchasing intention (*β* = 0.34, *p* < 0.01). Social norms were positively related to pro-domestic consumer ethnocentrism (*β* = 0.61, *p* < 0.01) and anti-foreign consumer ethnocentrism (*β* = 0.38, *p* < 0.01). Pro-domestic consumer ethnocentrism was positively related to domestic purchasing intention (*β* = 0.27, *p* < 0.01), while anti-foreign consumer ethnocentrism was not related to domestic purchasing intention (*β* = −0.07, *p* = 0.34). Social norms were positively related to domestic product judgments (*β* = 0.30, *p* < 0.01). Domestic product judgments were positively related to domestic purchasing intention (*β* = 0.21, *p* < 0.01). Pro-domestic consumer ethnocentrism was positively related to domestic product judgments (*β* = 0.29, *p* < 0.01), while anti-foreign consumer ethnocentrism was not related to domestic product judgments (*β* = 0.09, *p* = 0.26).

### 4.5. Mediation Analysis

To test the mediating effects, we performed a bootstrapping procedure with 5000 samples and a 95% confidential interval (CI) with Mplus 7.4, and the results are presented in Table 6. If the CI contains a value of 0, the effects are not significant, and if the CI does not contain a value of 0, the effects are significant. We found that the direct effect of social norms on domestic purchasing intention was significant (effect = 0.40, 95% CI = 0.22 to 0.56). The indirect effect of pro-domestic consumer ethnocentrism (effect = 0.20, 95% CI = 0.05 to 0.34), the indirect effect of domestic product judgments (effect = 0.07, 95% CI = 0.03 to 0.15), and the serial indirect effect of pro-domestic consumer ethnocentrism and domestic product judgments (effect = 0.04, 95% CI = 0.01 to 0.10) on the relationship between social norms and domestic purchasing intention were significant. The indirect effect of anti-foreign consumer ethnocentrism (effect = −0.03, 95% CI = −0.11 to 0.03) and the serial indirect effect of anti-foreign consumer ethnocentrism and domestic product judgments (effect = 0.01, 95% CI = 0.00 to 0.03) were not significant. Above all, H1, H2, H3, and H4 were all supported.

## 5. Discussion

The current study reveals that social norms affect domestic purchasing intention through four paths, i.e., the direct path, motivational path, cognitive path, and motivational–cognitive path. In addition, this research shows that consumer ethnocentrism has two dimensions, i.e., pro-domestic and anti-foreign consumer ethnocentrism, and they function differently in the model.

Specifically, this research shows that social norms are positively related to domestic purchasing intention, and their direct effect on domestic purchasing intention is significant, which is consistent with H1 and previous studies [9,10,11]. The results support the theory of planned behavior, showing that social norms are important predictors of people’s behavioral intentions [19].

Social norms are positively related to pro-domestic consumer ethnocentrism and pro-domestic consumer ethnocentrism is positively related to domestic purchasing intention. The mediating effect of pro-domestic consumer ethnocentrism is significant, while it is not significant for anti-foreign consumer ethnocentrism, which is consistent with H2 and previous studies [38,39,40]. These results support social cognition theory and norm activation theory, i.e., people will internalize social norms as their personal norms (pro-domestic consumer ethnocentrism) through social learning, and personal norms are the direct predictor of people’s behavioral intention [30,33]. The results further validate norm activation theory, which indicates that social norms can impact behavioral intention indirectly through personal norms [66]. The “social norms-personal norms-consumer behavior” link has been tested in many fields, such as organic food consumption [26,31,32] and fair trade products consumption [25]. This research is the first to apply this theory to domestic product consumption.

Social norms are positively related to domestic product judgments, and domestic product judgments are positively related to domestic purchasing intention. Domestic product judgments play a significant mediating role. The results are consistent with H3 and previous studies [41,47], supporting attribution theory and the hypothesis of the economic man, i.e., people will attribute the fact that most people buy domestic products to their high quality to some extent, and better product judgments lead to higher purchasing intention [44].

Pro-domestic consumer ethnocentrism is positively related to domestic product judgments. The two factors have a sequential mediating effect on the relationship between social norms and domestic purchasing intention, while anti-foreign consumer ethnocentrism does not. These results are consistent with H4 and previous studies [52,56], supporting social identity theory, i.e., people who are identified with a group will have an in-group preference [22]. The results confirm that motivations influence cognitions and these factors function together in consumer behavior [47,67].

The current research shows that consumer ethnocentrism has two dimensions in China, namely, pro-domestic and anti-foreign consumer ethnocentrism. The mean score of the former is higher than midpoint 4, while the mean score of the latter is lower than 4. These results confirm previous studies conducted in China and are consistent with the situations in other countries, including Azerbaijan and Greece [5,55,59]. Moreover, this research finds that pro-domestic consumer ethnocentrism has a great impact on domestic product judgments and domestic purchasing intention, while anti-foreign consumer ethnocentrism does not. This is because anti-foreign consumer ethnocentrism solely represents a negative attitude towards purchasing foreign products, which does not necessarily imply a positive attitude towards buying domestic ones. Domestic purchasing intention can be influenced by numerous other factors. Hence, even if consumers hold negative attitudes towards foreign products, they will still take into account other factors to make their purchasing decisions. The different effects of the two dimensions of consumer ethnocentrism on domestic product judgments and domestic purchasing intention are consistent with previous studies [56,58].

### 5.1. Contributions and Implications

The current study has theoretical contributions. Firstly, it is the first to explore the mechanisms of social norms’ effect on domestic purchasing intention, making up for the lack of empirical results and expanding the application of related theories, such as norm activation theory; secondly, it is the first to investigate the effects of the two dimensions of consumer ethnocentrism on domestic product judgments and domestic purchasing intention at the same time, and it is the first to explore the effects of the two dimensions of consumer ethnocentrism on domestic product judgments in China, increasing the knowledge on Chinese consumers and enriching the literature on consumer ethnocentrism.

This research has practical contributions. Firstly, it shows a significant direct effect of social norms on domestic purchasing intention. This suggests that administrators can cultivate pro-domestic social norms by issuing pro-domestic policies or using celebrity endorsement to promote people’s domestic purchasing intention. Secondly, it indicates a significant mediating effect of pro-domestic consumer ethnocentrism, instead of anti-foreign consumer ethnocentrism. This reminds us to pay attention to the internalization of social norms and to distinguish the different dimensions of consumer ethnocentrism. Appropriate policies should be set up to encourage people to support domestic products without excluding foreign ones, which will benefit both domestic and international economies. Thirdly, it demonstrates a significant mediating effect of domestic product judgments, suggesting that more attention should be paid to improving the quality of domestic products and expanding the influence of domestic brands.

### 5.2. Limitations and Future Studies

The current research is not without limitations. Firstly, it uses cross-sectional data, which can only claim correlational relationships. Future studies can conduct experiments to indicate causal relationships. Secondly, it adopts a general construct of social norms, which cannot distinguish the effects of different types. Future studies can divide social norms into pro-domestic social norms and anti-foreign social norms, or descriptive social norms and injunctive social norms to obtain more specific results. Thirdly, it measures purchasing intention only. Future studies can measure real purchasing behavior to obtain more concrete conclusions. Fourthly, it uses a convenience sample in China, which limits the generalizability of the results to some extent. Future studies can verify the relationships in other samples or other cultures to improve the external validity of the current findings.

## 6. Conclusions

The current study reveals that social norms affect domestic purchasing intention through four paths, i.e., the direct path, motivational path, cognitive path, and motivational–cognitive path. Consumer ethnocentrism and domestic product judgments, as the motivational factor and the cognitive factor, respectively, play mediating and serial mediating roles in the relationship between social norms and domestic purchasing intention. In addition, consumer ethnocentrism has two dimensions, i.e., pro-domestic and anti-foreign consumer ethnocentrism, and only the former plays a significant role in the model. This research has theoretical contributions to research on domestic purchasing intention and practical implications for interventions in domestic purchasing behavior.

## Figures and Tables

**Table 1:** Demographic profile of the respondents.

Demographic	Group	Frequency	Percentage
Gender	1. Male	159	46.0%
	2. Female	187	54.0%
Age	1. 18–25	126	36.4%
	2. 26–35	82	23.7%
	3. 36–45	63	18.2%
	4. 46–55	40	11.6%
	5. Above 55	35	10.1%
Education level	1. Elementary school or below	5	1.4%
	2. Junior high school	18	5.2%
	3. Senior high school	50	14.5%
	4. College	53	15.3%
	5. Bachelor’s degree	184	53.2%
	6. Master’s/Doctor’s degree	36	10.4%
Monthly Income	1. Less than CNY 1000	31	9.0%
	2. CNY 1001–2000	32	9.2%
	3. CNY 2001–3000	31	9.0%
	4. CNY 3001–4000	45	13.0%
	5. CNY 4001–6000	84	24.3%
	6. CNY 6001–8000	66	19.1%
	7. CNY 8001–10,000	24	6.9%
	8. Above CNY 10,000	33	9.5%

Note: the exchange rate for Chinese Yuan (CNY) to the US Dollar (USD) is around 7 CNY to 1 USD in May 2023.

**Table 2:** Items, descriptive statistics, and factor loadings of the constructs.

Constructs	Items	*M*	*SD*	FL
Social norms	1. In the current society, people generally agree that we should support domestic products.	5.89	1.06	0.70
	2. In the current society, most people think that buying Chinese products is a glorious thing.	5.67	1.22	0.70
	3. In the current society, people consciously choose to buy domestic products.	5.54	1.20	0.74
Pro-domestic consumer ethnocentrism	1. Chinese people should always buy Chinese-made products instead of imports. (CE1)	4.68	1.74	0.83
	2. Only those products that are unavailable in China should be imported. (CE2)	4.35	1.75	0.69
	3. Chinese products, first, last, and foremost. (CE4)	5.44	1.49	0.74
	4. A real Chinese should always buy Chinese-made products. (CE7)	4.59	1.75	0.82
	5. We should purchase products manufactured in China instead of letting other countries get rich off us. (CE8)	4.70	1.71	0.82
	6. It is always best to purchase Chinese products. (CE9)	4.70	1.80	0.83
	7. It may cost me in the long run, but I prefer to support Chinese products. (CE13)	4.99	1.51	0.71
Anti-foreign consumer ethnocentrism	1. Purchasing foreign-made products is un-Chinese. (CE5)	3.22	1.75	0.83
	2. It is not right to purchase foreign products, because it puts Chinese out of jobs. (CE6)	3.30	1.75	0.85
	3. Chinese should not buy foreign products, because this hurts Chinese business and causes unemployment. (CE11)	3.83	1.85	0.86
	4. Curbs should be put on all imports. (CE12)	2.79	1.81	0.74
	5. Foreigners should not be allowed to put their products on our markets. (CE14)	3.32	1.84	0.87
	6. Foreign products should be taxed heavily to reduce their entry into China. (CE15)	4.01	1.89	0.85
	7. We should buy from foreign countries only those products that we cannot obtain within our own country. (CE16)	4.10	1.78	0.73
	8. Chinese consumers who purchase products made in other countries are responsible for putting their fellow Chinese out of work. (CE17)	3.82	1.82	0.86
Domestic product judgments	1. The products made in China are friendly to the common people.	5.66	1.23	0.71
	2. Chinese products always put consumers’ interests first.	5.01	1.58	0.81
	3. The quality and performance of products made in China are excellent.	5.24	1.37	0.88
	4. Chinese products can always lead the trend of consumers.	4.90	1.54	0.85

Note: FL = factor loading.

**Table 3:** Descriptive statistics, reliability, validity, and correlations of the constructs.

Variables	*M*	*SD*	*α*	CR	AVE	1	2	3	4	5
1. Social norms	5.70	0.95	0.76	0.76	0.51	(0.71)				
2. Pro-domestic consumer ethnocentrism	4.78	1.36	0.91	0.92	0.61	0.61 **	(0.78)			
3. Anti-foreign consumer ethnocentrism	3.55	1.53	0.94	0.94	0.68	0.38 **	0.80 **	(0.82)		
4. Domestic product judgments	5.20	1.24	0.88	0.89	0.66	0.51 **	0.54 **	0.43 **	(0.81)	
5. Domestic purchasing intention	5.87	1.13	----	----	----	0.58 **	0.55 **	0.38 **	0.49 **	----

Note: ** *p* < 0.01. CR = composite reliability; AVE = average variance extracted. Numbers in parentheses represent the square roots of AVE.

**Table 4:** Heterotrait–monotrait (HTMT) ratios.

Variables	1	2	3	4
1. Social norms				
2. Pro-domestic consumer ethnocentrism	0.86			
3. Anti-foreign consumer ethnocentrism	0.61	0.47		
4. Domestic product judgments	0.74	0.45	0.62	

**Table 5:** Results of structural model.

The Model Paths	*β*	*SE*	*t*	*p*
Social norms → domestic purchasing intention	0.34 **	0.07	4.80	0.00
Social norms → pro-domestic consumer ethnocentrism	0.61 **	0.04	17.06	0.00
Social norms → anti-foreign consumer ethnocentrism	0.38 **	0.05	8.19	0.00
Pro-domestic consumer ethnocentrism → domestic purchasing intention	0.27 **	0.10	2.65	0.01
Anti-foreign consumer ethnocentrism → domestic purchasing intention	−0.07	0.08	−0.96	0.34
Social norms → domestic product judgments	0.30 **	0.07	4.11	0.00
Domestic product judgments → domestic purchasing intention	0.21 **	0.07	2.99	0.00
Pro-domestic consumer ethnocentrism → domestic product judgments	0.29 **	0.10	3.06	0.00
Anti-foreign consumer ethnocentrism → domestic product judgments	0.09	0.08	1.14	0.26

Note: ** *p* < 0.01; “not significant” means that the effect would be not significant.

**Table 6:** Results of mediation analysis.

The Mediation Paths	Effect	SE	LLCI	ULCI	Decision
Direct effect					
Social norms → domestic purchasing intention	0.40	0.09	0.22	0.56	Support H1
Indirect effect					
Social norms → pro-domestic CE → domestic purchasing intention	0.20	0.08	0.05	0.34	Support H2
Social norms → anti-foreign CE → domestic purchasing intention	−0.03	0.04	−0.11	0.03	
Social norms → domestic product judgments → domestic purchasing intention	0.07	0.03	0.03	0.15	Support H3
Social norms → pro-domestic CE → domestic product judgments → domestic purchasing intention	0.04	0.02	0.01	0.10	Support H4
Social norms→ anti-foreign CE → domestic product judgments→ domestic purchasing intention	0.01	0.01	0.00	0.03	

Note: CE = consumer ethnocentrism; LLCI = bootstrapping lower-level confidential interval; ULCI = bootstrapping upper-level confidential interval; “not significant” means that the effect would be not significant.

## Data Availability

The datasets used and/or analyzed during the current study are available from the corresponding author upon reasonable request.
